# DNA damage-induced activation of ATM promotes β-TRCP-mediated Mdm2 ubiquitination and destruction

**DOI:** 10.18632/oncotarget.640

**Published:** 2012-09-11

**Authors:** Zhiwei Wang, Hiroyuki Inuzuka, Jiateng Zhong, Hidefumi Fukushima, Lixin Wan, Pengda Liu, Wenyi Wei

**Affiliations:** ^1^ Department of Pathology, Beth Israel Deaconess Medical Center, Harvard Medical School, Boston, MA; ^2^ Department of Pathophysiology, Norman Bethune College of Medicine, Jilin University, Changchun, P. R. China

**Keywords:** ATM, CKIδ, Mdm2, β-TRCP, ubiquitination, destruction, DNA damage, cancer

## Abstract

The Mdm2 oncoprotein promotes p53 ubiquitination and destruction. Yet, exact molecular mechanisms of Mdm2 destruction itself, under DNA damaging conditions, remain unclear. Recently, we identified SCF^β-TRCP^ as a novel E3 ligase that targets Mdm2 for ubiquitination and destruction in a Casein Kinase Iδ (CKIδ)-dependent manner. However, it remains elusive how the β-TRCP/CKIδ/Mdm2 signaling axis is regulated by DNA damage signals to govern p53 activity. Consistent with previous studies, we found that inactivation of the Ataxia Telangiectasia Mutated (ATM) kinase, in turn, impaired DNA damage-induced Mdm2 destruction. Although phosphorylation of Mdm2 at Ser395 (an ATM phosphorylation site) facilitated Mdm2 interaction with β-TRCP, Ser395A-Mdm2 was degraded non-distinguishably from WT-Mdm2 by SCF^β-TRCP^ upon DNA damaging treatments. This indicates that in addition to phosphorylating Mdm2 at Ser395, ATM may govern Mdm2 stability through other unknown mechanisms. We further demonstrated that DNA damage-induced activation of ATM directly phosphorylated CKIδ at two well-conserved S/TQ sites, which promotes CKIδ nuclear localization to increase CKIδ-mediated phosphorylation of Mdm2, thereby facilitating subsequent Mdm2 ubiquitination by SCF^β-TRCP^. Our studies provide a molecular mechanism of how ATM could govern DNA damage-induced destruction of Mdm2 in part by phosphorylating both Mdm2 and CKIδ to modulate SCF^β-TRCP^–mediated Mdm2 ubiquitination. Given the pivotal role of Mdm2 in the negative regulation of p53, this work will also provide a rationale for developing CKIδ or ATM agonists as anti-cancer agents.

## INTRODUCTION

Human cancer development has been characterized to be a multi-step process that is associated with both activation of multiple oncogenes and inactivation of various tumor suppressor genes [1]. The p53 gene is considered as one of the prototypical tumor suppressor gene, as abnormalities of the p53 gene have been observed in more than half of all human cancers [2]. The p53 tumor suppressor protein plays a critical role in anti-proliferation activity through inducing cell cycle arrest, senescence, and programmed cell death [3]. Additionally, p53 has been reported to have a physiological function in regulating cellular responses to DNA damage in part by inducing either G1 or G2 checkpoints, allowing sufficient time for cells to repair their damaged DNA [4]. Recent studies have also shown that p53 is involved in governing the cellular apoptotic pathway under conditions when cells are exposed to excessive DNA damage beyond the ability of cells to efficiently repair them [4]. Given the pivotal roles of p53 in various cellular processes, p53 activity is tightly regulated at multiple levels including transcription, translation and protein stability [5]. Although several E3 ubiquitin ligases including Mdm2 [6], E6AP [7], TOPORS [8], RFFL [9], Cop1 [10] and Pirh2 [11] have been implicated in regulating p53 stability, mouse genetic studies strongly indicate that Mdm2 (mouse double minute 2) [12, 13] is the major negative regulator for p53 stability [6]. Consistent with the critical role of Mdm2 as the major physiological E3 ligase in promoting p53 destruction, the Mdm2 oncoprotein is frequently found to be overexpressed in a wide variety of human cancers [14]. Furthermore, mutations in the Mdm2 oncoprotein that disrupt ribosomal protein (RP)-mediated suppression of Mdm2 E3 ligase activity have been observed in human cancers [15]. In line with the critical role for Mdm2-mediated degradation of p53 in favoring tumorigenesis, a specific Mdm2 inhibitor (MI-219) has been reported to synergize with oxaliplatin, which inhibits DNA synthesis, to exhibit superior anti-cancer effects in solid tumors types bearing wild-type p53 [16].

However, the exact molecular mechanisms underlying Mdm2 overexpression in human cancers remain largely elusive. We recently identified that both cell cycle regulated and DNA damage-induced turnover of Mdm2 is controlled by SCF^β-TRCP^-mediated ubiquitination of Mdm2 [17, 18]. We further defined a critical role for Casein Kinase Iδ (CKIδ) in SCF^β-TRCP^-mediated degradation of Mdm2 [17, 18]. Importantly, depletion of β-TRCP resulted in attenuated p53 activity, reduced pulsatile waves of p53 in response to persistent DNA damage, and increased resistance to DNA damage-induced apoptosis [18]. However, it remains largely uncharacterized how DNA damaging signals control β-TRCP-mediated Mdm2 destruction. Furthermore, even though CKIδ kinase was identified to play a critical role in mediating cell cycle-dependent destruction of Mdm2 by SCF^β-TRCP^ [17, 18], it is not fully understood whether under the DNA damage conditions, the CKIδ kinase is activated to govern the Mdm2/p53 pathway.

To this end, multiple studies have demonstrated that the ATM (ataxia telangiectasia mutated) tumor suppressor protein can regulate Mdm2 under genotoxic stresses [19-21]. ATM is one of the founding members of the PIKK (phosphatidyl inositol 3-kinase related kinase) family of kinases [22]. In response to DNA damage caused by UV exposure or reactive oxygen species (ROS), ATM is activated, which subsequently leads to the phosphorylation of p53. ATM-mediated phosphorylation of p53 disrupts the binding between Mdm2 and p53, resulting in enhanced p53 stability by escaping Mdm2-mediated ubiquitination [20]. In addition to p53, Mdm2 was also reported to undergo ATM-dependent phosphorylation on the Serine 395 (Ser395) residue in response to DNA damage [23]. Importantly, transgenic mice studies revealed that ATM phosphorylation of Mdm2 at Ser394 (equivalent to human Ser395 site) is required for p53 stabilization and activation after DNA damage [24]. These studies prompted us to further study whether the ATM kinase plays a critical role in SCF^β-TRCP^-mediated Mdm2 proteolysis following DNA damaging signals.

In the present study, for the first time, we report that ATM indeed participates in β-TRCP-mediated Mdm2 ubiquitination and destruction. Moreover, our results demonstrate that phosphorylation of Mdm2 by ATM at Ser395 is not necessary for β-TRCP-mediated Mdm2 ubiquitination after DNA damage. On the other hand, ATM-dependent phosphorylation of CKIδ promotes CKIδ nuclear localization to trigger CKIδ-mediated Mdm2 phosphorylation and subsequent destruction. Hence, our current study supports the critical role of ATM in SCF^β-TRCP^–mediated Mdm2 degradation. Furthermore, our results suggest that ATM agonists could be used as anti-cancer agents, in part by accelerating Mdm2 destruction.

## RESULTS

### Mdm2 E3-ligase activity is not required for DNA damage-induced Mdm2 destruction

Consistent with previous reports [18], we found that in multiple cell lines, the Mdm2 oncoprotein became more unstable following DNA damaging agent, such as etoposide treatment (Supplemental Figures 1A-B). Studies from multiple laboratories have indicated that DNA-damage could possibly augment Mdm2 auto-degradation [25, 26]. However, a recent study demonstrated that the E3-ligase activity of Mdm2 might not be required for its destruction [27]. To solve this potential discrepancy, we continued to explore whether a Ring-finger mutant Mdm2^C464A^ that is defective in its E3 ubiquitin ligase activity, also became unstable upon DNA damaging treatments. In line with previous reports [18, 27], we found that Mdm2^C464A^ becomes unstable after treatment with a variety of DNA damaging agents including 5-FU, Doxorubicin, Actinomycin D (Supplemental Figures 1C-E). These results suggest that E3 ligase(s) other than Mdm2 itself, such as recently identified β-TRCP [18], might govern Mdm2 destruction under DNA damage conditions. However, mechanistically, it remains largely unknown how DNA damaging signals are transduced to possibly control β-TRCP-mediated ubiquitination of Mdm2. As most β-TRCP substrates require prior-phosphorylation for recognition by β-TRCP [28], it is critical to reveal the upstream signaling pathway(s) that are activated by DNA damage to trigger β-TRCP-mediated timely destruction of Mdm2.

### ATM is involved in DNA damage-induced Mdm2 destruction

**Figure 1:**
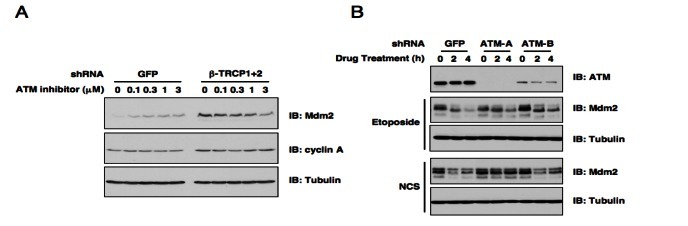
ATM is involved in DNA damage-induced Mdm2 destruction (A) Immunoblot analysis of HeLa cells transfected with the indicated shRNA constructs. Cells were treated with the indicated concentrations of the ATM inhibitor for 12 hours before harvesting. (B) Endogenous ATM expression was depleted in U2OS cells by infecting with the indicated lentiviral shRNA constructs (with shGFP as a negative control) and selected with 1 μg/ml puromycin to eliminate the non-infected cells. The resulting U2OS cell lines were treated with etoposide or neocarzinostatin (NCS) for the indicated durations to induce Mdm2 destruction before harvesting for immunoblot analysis.

Given the pivotal role of ATM in DNA damage response, we started our investigation by examining whether ATM participates in Mdm2 stability control. In keeping with our previous report [18], depletion of both isoforms of endogenous β-TRCP (β-TRCP1 and β-TRCP2) led to a significant increase of Mdm2 protein abundance (Figure 1A). More interestingly, inactivation of ATM kinase by its pharmaceutical inhibitor [16, 29], led to a dose-dependent increase in Mdm2 abundance as well in shGFP-treated control HeLa cells (Figure 1A). However, ATM inhibitor could not induce Mdm2 expression in shβ-TRCP-treated HeLa cells, indicating that ATM might govern Mdm2 stability in a β-TRCP-dependent manner. Notably, consistent with previous reports [30-32], depletion of endogenous ATM with multiple independent shRNA vectors moderately affected Mdm2 destruction in response to various DNA damaging agents (Figure 1B). These results indicate a possible role for the ATM signaling pathway in regulating DNA damage-induced Mdm2 destruction. The next critical question we intended to address is to determine which is the major downstream signaling route through which DNA damage-induced activation of ATM influences Mdm2 stability.

### ATM-mediated phosphorylation of Mdm2 is not critical for Mdm2 destruction

**Figure 2:**
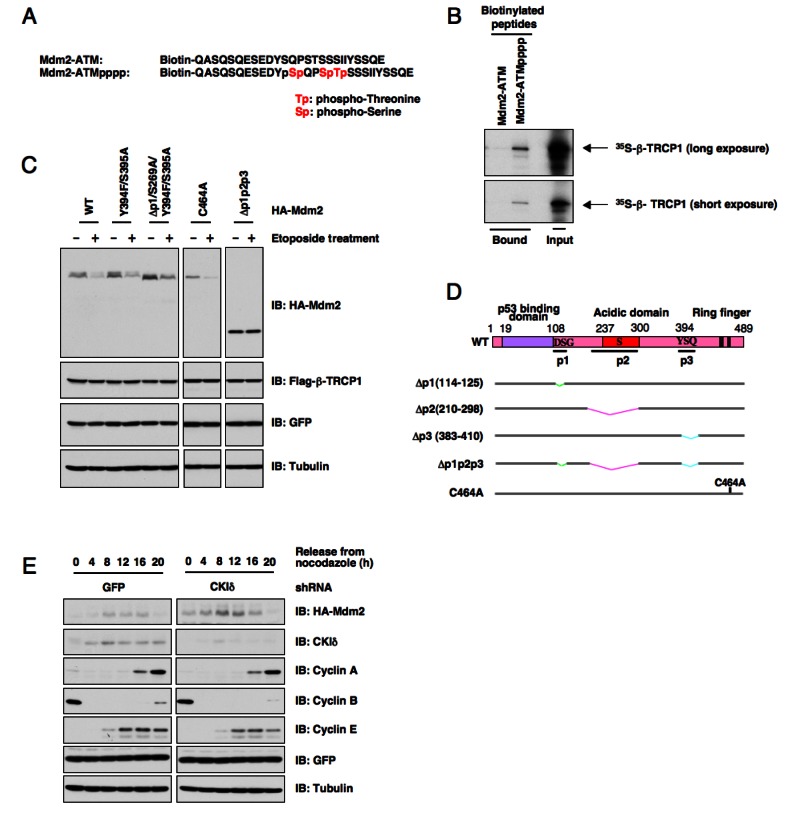
ATM-mediated phosphorylation of Mdm2 is not critical for Mdm2 destruction (A) Schematic illustration of the biotinylated Mdm2 peptides used in this study. (B) Autoradiography of ^35^S-labeled β-TRCP1 bound to the indicated biotinylated peptides. (C) Casein Kinase Iδ phosphorylates Mdm2 at multiple sites to trigger Mdm2 destruction mediated by β-TRCP1. Immunoblot analysis of U2OS cells transfected with the indicated HA-Mdm2 and Flag-β-TRCP1 plasmids. A plasmid encoding GFP was used as a negative control for transfection efficiency. Thirty hours post-transfection, 25 μM etoposide was used for up to three hours to trigger Mdm2 destruction before harvesting cells for immunoblots. (D) Schematic illustration of the various Mdm2 deletion mutants. p1, p2 and p3 are three identified PEST-sequence containing motifs that contain the identified CKI-phosphorylation sites. Loss of p1, p2, p3 creates a non-degradable mutant of Mdm2 (Δp1p2p3). C464A mutant form of Mdm2 lacks the E3 ubiquitin ligase activity, thus is defective in undergoing self-ubiquitination. (E) Immunoblot analysis of 293T cells transfected with HA-Mdm2 and the indicated shRNA constructs, after synchronization with nocodazole and release.

As Mdm2 has been reported to be phosphorylated by ATM at the Ser395 site, we continued our study by examining whether direct phosphorylation of Mdm2 by ATM affects Mdm2 interaction with its E3 ligase, β-TRCP1. Using immobilized biotinylated Mdm2 peptides derived from the range spanning the identified ATM phosphorylation site (Ser395) (Figure 2A), we found that a phosphorylation event occurring at Ser395 could trigger Mdm2 interaction with β-TRCP1 (Figure 2B). It is thus possible that ATM-phosphorylation on Ser395 in Mdm2 may prime for the subsequent CKI-mediated phosphorylation. However, we found that mutation of the Ser395 site did not affect etoposide-induced Mdm2 destruction (Figures 2C-D). Furthermore, overexpression of ATM alone failed to significantly trigger Mdm2 destruction, or to promote Mdm2 ubiquitination *in vitro* (data not shown), indicating that ATM may require other kinase(s) to synergistically regulate Mdm2 degradation. Since our previous studies identified CKIδ as an upstream kinase responsible for Mdm2 destruction (Figure 2E) [18], it was thus of great interest for us to further investigate whether ATM promotes Mdm2 degradation mainly by influencing CKIδ-mediated phosphorylation of Mdm2.

### ATM phosphorylates CKIδ to possibly regulate its ability to interact with, and subsequently promote Mdm2 destruction

**Figure 3:**
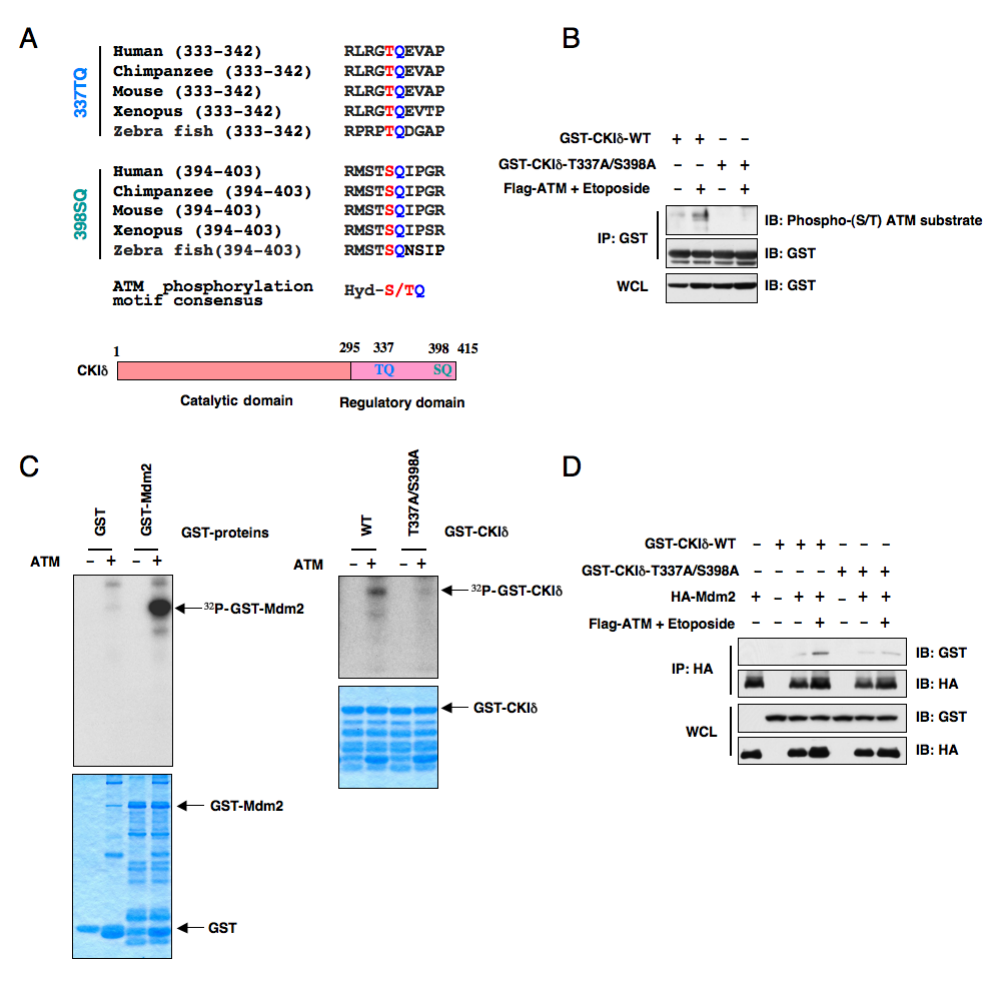
ATM phosphorylates Casein Kinase Iδ to regulate its subcellular localization and its ability to interact with Mdm2 (A) Protein sequence illustration of the two putative ATM phosphorylation sites present in the C-terminus of CKIδ across different species. Hyd: hydrophobic amino acids. (B) Immunoblot analysis (IB) of whole cell lysates (WCL) and immunoprecipitates (IP) derived from 293T cells transfected with the indicated GST-CKIδ constructs in the presence or absence of Flag-ATM. Where indicated, the ATM kinase was activated by treatment with etoposide for 30 minutes before harvesting. The phosphorylation status of CKIδ by ATM *in vivo* was detected with a specific phospho-(S/T) ATM substrate antibody. (C) ATM phosphorylates both Mdm2 and CKIδ *in vitro*. The ATM kinase was purified by Flag-immunoprecipitation from 293T cells, and then incubated with 5 μg of GST-Mdm2 or the indicated GST-CKIδ proteins (with GST protein as a negative control) in the presence of γ-^32^P-ATP. The kinase reaction products were resolved by SDS-PAGE and phosphorylation was detected by autoradiography. (D) Immunoblot (IB) analysis of whole cell lysates (WCL) and immunoprecipitates (IP) derived from 293T cells transfected with HA-Mdm2 and the indicated GST-CKIδ constructs. Thirty hours post-transfection, cells were pretreated with 10 μM MG132 for 2 hours to block the proteasome pathway and then treated with 25 μM etoposide (or DMSO as control) for 1.5 hours before harvesting.

Although CKI has been reported to be activated by DNA damage in Drosophila cells [33], the exact molecular mechanisms of how CKI is activated following DNA damaging treatment in human cells are largely unknown. Upon subsequent examination of the CKIδ protein sequence, we found two well-conserved potential ATM phosphorylation [34] sites in its regulatory domain (Figure 3A). In keeping with this finding, we found that ATM could phosphorylate CKIδ (Figures 3B-C) to potentially regulate its ability to interact with Mdm2 (Figure 3D). As a result, etoposide treatment, which activates ATM kinase, promotes CKIδ and Mdm2 interaction while the CKIδ mutant (CKIδ-T337A/S398A), where the two conserved ATM sites were inactivated, failed to interact with Mdm2 upon etoposide treatment (Figure 3D).

### ATM-mediated phosphorylation of CK1δ promotes CK1δ nuclear localization

**Figure 4:**
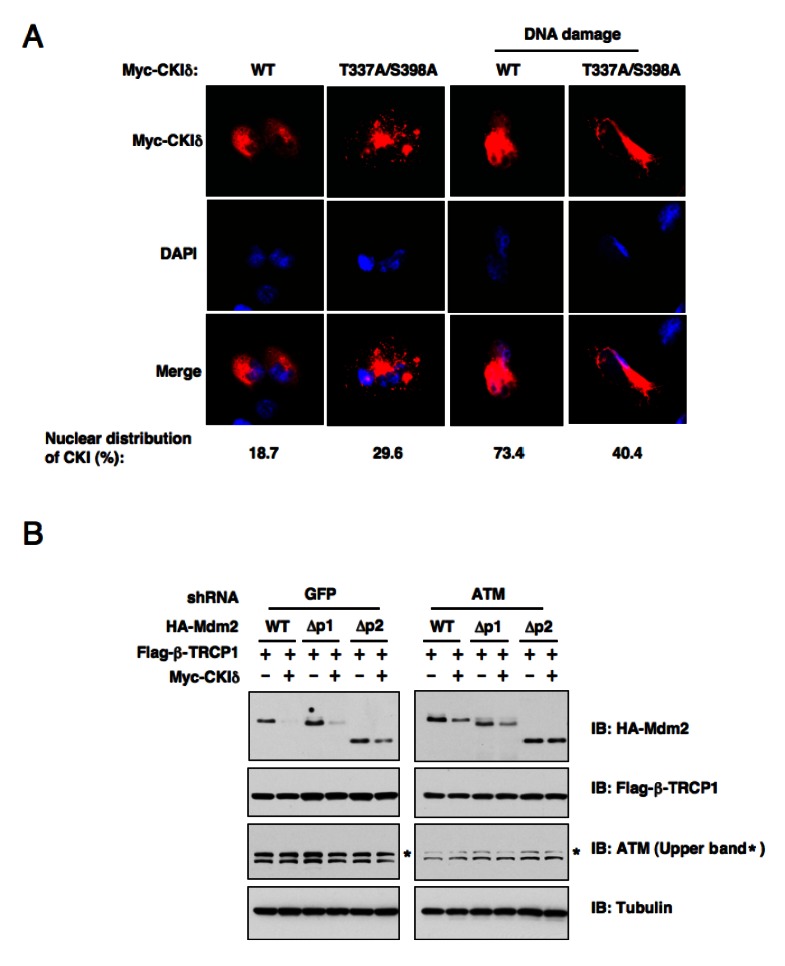
ATM-mediated phosphorylation of CK1δ promotes CK1δ localization, and subsequent phosphorylation of Mdm2 to trigger Mdm2 degradation (A) ATM-mediated phosphorylation of CK1δ promotes CK1δ nuclear localization. Immunofluorescence and DAPI staining of U2OS cells transfected with the indicated Myc-CKIδ constructs. Cells were treated with or without 10μM doxorubicin for 1 hour before fixation. (B) Stability of the Mdm2 protein is controlled by ATM. U2OS cells were infected with the indicated lentiviral shRNA construct and selected with 1 μg/ml puromycin to eliminate the non-infected cells. The resulting U2OS cell lines were transfected with the indicated HA-Mdm2, Flag-β-TRCP1 and Myc-CKIδ constructs. Immunoblots were performed to monitor the changes of HA-Mdm2. p1 and p2 are two of the three identified major PEST-sequence containing motifs that contain the identified CKI-phosphorylation sites.

To further understand the molecular mechanisms underlying how DNA damaging signals may potentially regulate CKIδ activity, we immunoprecipitated CKIδ from U2OS cells after treatment with etoposide for the indicated time periods to measure its kinase activity. The CKIδ activity changes were indicated by its ability to phosphorylate recombinant Skp2 or CKIδ itself *in vitro*. Surprisingly, we only detected a moderate increase in the intrinsic CKIδ kinase activity in response to DNA damage (Supplemental Figures S2A-B). These results indicate that rather than directly regulating the CKIδ kinase activity, DNA damaging signals might influence the association of CKIδ with its substrates including Mdm2 by controlling CKIδ cellular localization. In support of this notion, we found that in agreement with a previous report [35], CKIδ mainly resides in the cytoplasm in non-stressed cells. However, in response to DNA damage, a significant fraction of endogenous CKIδ translocated into the nucleus to interact with, and modify, the Mdm2 protein (Figure 4A). More importantly, we showed that inactivation of the two putative ATM sites, which are located at the C-terminus of CKIδ, disrupts its ability to enter the nucleus following DNA damage (Figure 4A). This result strongly suggests that ATM plays an important role in DNA damage-induced nuclear translocation of CKIδ to promote CKIδ-mediated Mdm2 phosphorylation, which might influence the subsequent Mdm2 ubiquitination and destruction mediated by the β-TRCP/CKIδ signaling axis. Furthermore, in support of a critical role of ATM in β-TRCP/CKIδ−mediated Mdm2 destruction, we found that depletion of endogenous ATM attenuated the destruction of ectopically expressed WT-Mdm2, Δp1-Mdm2 or Δp2-Mdm2 after co-expression of both β-TRCP and CKIδ (Figure 4B). On the other hand, consistent with our previous report [18], depleting all possible β-TRCP-recognizable degrons containing the identified CKIδ phosphorylation sites created a non-degradable Mdm2 (Δp1p2p3-Mdm2) that resists the destruction effects mediated by co-expressing β-TRCP and CKIδ (data not shown). These results elucidated the specificity of this *in vivo* degradation assay, and further implicated that CKIδ-dependent phosphorylation events is the major signaling route through which DNA damage-dependent activation of ATM might control timely turnover of Mdm2 during genotoxic stress.

## DISCUSSION

**Figure 5:**
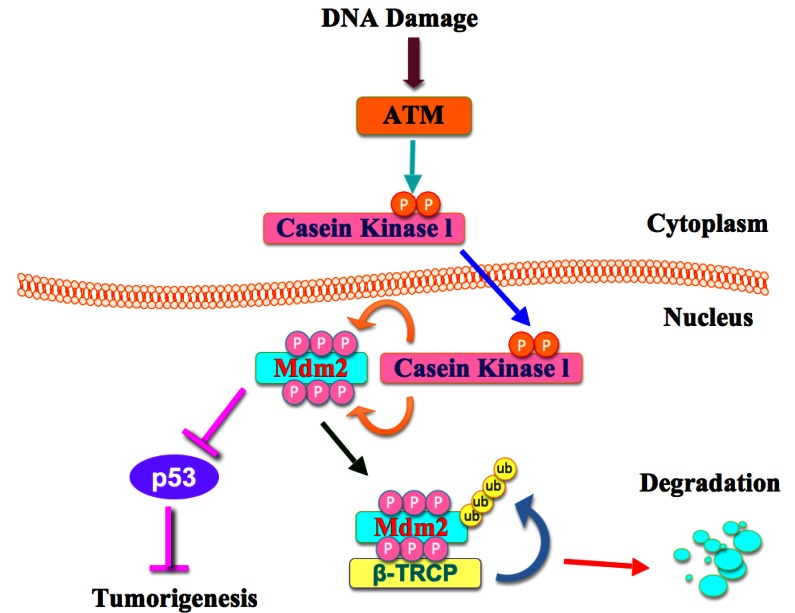
A proposed role for the ATM in β-TRCP-mediated Mdm2 ubiquitination and destruction

Cells have complex mechanisms to protect their genomes from various endogenous and exogenous stressors including UV exposure, γ-irradiation, ROS, and mutagens, which cause DNA damage [36]. For example, cells have multiple repair mechanisms and checkpoint responses to maintain their genomic stability. DNA damage responses are governed by multiple signaling pathways including the ATM-Chk2 and ATR-Chk1 pathways [37]. It has been reported that DNA damage leads to ATM-dependent USP7S (a specific isoform of USP7) dephosphorylation by PPM1G (protein phosphatase magnesium-dependent 1 gamma), thus resulting in the inactivation and degradation of USP7S to promote Mdm2 ubiquitination and degradation [38]. Although activation of ATM has been found to be crucial for DNA damage responses, the underlying molecular mechanisms are largely unknown. Here we report that DNA damage activates ATM, which subsequently phosphorylates CKIδ to promote its translocation into the nucleus where phosphorylation of Mdm2 by CKIδ ultimately triggers Mdm2 degradation by SCF^β-TRCP^ (Figure 5).

It has been shown that the inhibitory function of Mdm2 towards p53 plays a major role in modulating p53 activity following DNA damage, a process that is subjected to many layers of regulation [39]. Among them, the ATM/ATR/Chk kinase cascade induced by DNA damage has an important function in the regulation of p53 activity in part by directly phosphpryating p53 [40]. Inhibition of ATM resulted in p53 shift from the nuclear to cytosolic fractions, which inactivates its transcriptional activity [41]. Furthermore, ATM has been demonstrated to phosphorylate Mdm2 at Ser395 [23]. Mechanistically, ATM phosphorylation of Mdm2 inhibits the ability of Mdm2 to poly-ubiquitinate p53, thereby resulting in p53 stabilization [42]. This prompted us to explore whether ATM participates in Mdm2 stability control. Indeed, we found that the use of an ATM inhibitor increased Mdm2 abundance in control HeLa cells, but not in β-TRCP-depleted HeLa cells, suggesting that ATM could govern Mdm2 stability in a β-TRCP-dependent manner. However, inactivating Ser395 in Mdm2 did not significantly stabilize Mdm2 under DNA damaging conditions, arguing that ATM may control Mdm2 turnover by phosphorylating other critical components of the Mdm2 destruction pathway. In support of this notion, we found that in response to DNA damage, activation of the ATM kinase directly phosphorylates CKIδ at its C-terminus to regulate its subcellular localization and interaction with Mdm2 (Figure 3A). These data indicate that DNA damage primarily modulates the accessibility of CKIδ cellular localization (Figure 4A) rather than its kinase activity (Supplemental Figures S2A-B) to influence Mdm2 stability.

On the basis of our results, we propose a hypothetical model by which ATM is the upstream kinase that following activation by DNA damaging signals, could phosphorylate CKIδ to potentially regulate its subcellular localization and its ability to interact with and regulate Mdm2 (Figure 5). However, we recognize that ATM may also phosphorylate other unknown substrates to affect Mdm2 turnover. Therefore, further in-depth studies are needed to investigate the precise molecular mechanism of how ATM affects CKIδ cellular localization, as well as how ATM and CKIδ work synergistically to promote Mdm2 destruction following DNA damage. In summary, we presented experimental evidence that strongly supports the pivotal roles of ATM in β-TRCP-mediated Mdm2 degradation. Since ATM and CK1 synergistically control β-TRCP-mediated Mdm2 ubiquitination and destruction, our work provides a rational for the use of CKI and/or ATM agonists as anti-cancer agents to promote Mdm2 destruction, which might improve the overall survival of patients diagnosed with cancer in the future.

## MATERIALS AND METHODS

### Plasmids

HA-Mdm2 construct was kind gift from Dr. Jiandong Chen. Various Mdm2 and CKIδ mutants were generated using the QuikChange XL Site-Directed Mutagenesis Kit (Stratagene) according to the manufacturer's instructions. The C-terminus fragment (aa 285-415) of CKIδ was subcloned using the Pfu polymerase (Stratagene) into the pGEX vector to create GST-CKIδ in frame fusion proteins. Flag-β-TRCP1, shRNA-β-TRCP1+2, shTRCP1, shTRCP2, and shRNA-GFP constructs were described previously [43, 44]. Flag-ATM construct was obtained from Michael Kastan. shRNA constructs against various CKI isoforms were obtained from Dr. Jianping Jin. Lentiviral shRNA constructs against GFP, ATM and CKIδ were obtained from Dr. William Hahn.

### Antibodies and Reagents

Anti-cyclin A (SC-751), anti-cyclin B (SC-245), anti-cyclin E (SC-247), anti-Casein Kinase Iδ (R-19) (SC-6474) and polyclonal anti-HA (SC-805) antibodies were purchased from Santa Cruz. Polyclonal anti-FLAG (F2425), monoclonal anti-FLAG (F-3165), peroxidase-conjugated anti-mouse secondary antibody (A4416), peroxidase-conjugated anti-rabbit secondary antibody (A4914), and Anti-tubulin (T-5168) antibodies were purchased from Sigma. Monoclonal anti-HA antibody (MMS-101P) was purchased from Covance. The anti-GFP (632380) antibody was purchased from Invitrogen. The anti-phospho-Ser1981 ATM (4526) antibody was purchased from Cell Signaling. Anti-Mdm2 (Ab1:OP46) was purchased from Calbiochem. Oligofectamine, Lipofectamine and Plus reagents were purchased from Invitrogen.

### Immunoblots and Immunoprecipitation

Cells were lysed in EBC (50 mM Tris pH 8.0, 120 mM NaCl, 0.5% NP-40) buffer supplemented with protease inhibitors (Complete Mini, Roche) and phosphatase inhibitors (phosphatase inhibitor cocktail set I and II, Calbiochem). The protein concentrations of the lysates were measured using the Bio-Rad protein assay reagent on a Beckman Coulter DU-800 spectrophotometer. The lysates were then resolved by SDS-PAGE and immunoblotted with indicated antibodies. For immunoprecipitation, 800 μg lysates were incubated with the appropriate antibody (1-2 g) for 3-4 hours at 4 C followed by one hour-incubation with Protein A sepharose beads (GE Healthcare). Immuno-complexes were washed five times with NETN buffer (20 mM Tris, pH 8.0, 100 mM NaCl, 1 mM EDTA and 0.5% NP-40) before being resolved by SDS-PAGE and immunoblotted with indicated antibodies.

### *In vitro* Kinase Assay

293T cells were transfected with Flag-ATM constructs. Forty-eight hours later, ATM was immunoprecipitated using Flag-matrix (Sigma). Afterwards, it was incubated with 5 μg of indicated GST fusion proteins in the presence of 5 μCi [γ-^32^P] ATP and 20 μM cold ATP in the ATM kinase reaction buffer for 15-30 minutes. The reaction was stopped by the addition of SDS-containing lysis buffer, resolved on SDS-PAGE, and detected by autoradiography. Casein Kinase Iδ was purchased from New England Biolabs. The Casein Kinase I *in vitro* kinase assay was performed according to the manufacturer's instructions (New England Biolabs). Briefly, 5 μg of indicated GST fusion proteins were incubated with purified active Casein Kinase I in the presence of 5 μCi [γ-^32^P] ATP and 200 μM cold ATP in the Casein Kinase I reaction buffer for 15-30 minutes. The reaction was stopped by the addition of SDS-containing lysis buffer, resolved on SDS-PAGE, and detected by autoradiography.

### Immunofluorescence Microscopy

Cells were fixed with 4% paraformaldehyde for 10 min, permeabilized in 0.5% Triton X-100 for 10 min, and incubated in PBS and 10% goat serum blocking solution for 1 h. The cells were incubated for 2 h with anti-CK1δ in 5% goat serum and were stained for 1 h with Alexa Fluor 594-conjugated secondary antibody (1:500). The slides were mounted with mounting medium containing antifade reagent and 4,6-diamidino-2-phenylindole. Cells were viewed under fluorescence microscope.

## Supplementary Figures


