# Supplementary material for: DNA damage-induced activation of ATM promotes β-TRCP-mediated Mdm2 ubiquitination and destruction
Source: Oncotarget. 2012 Sep 11;3(9):1026–35. doi: 10.18632/oncotarget.640 (PMC3660052; doi:10.18632/oncotarget.640)
Supplement: Supplementary file 1 [file oncotarget-03-1026-s001.pdf]

DNA damage-induced activation of ATM promotes β-TRCP-mediated Mdm2 ubiquitination and destruction – Wang et al

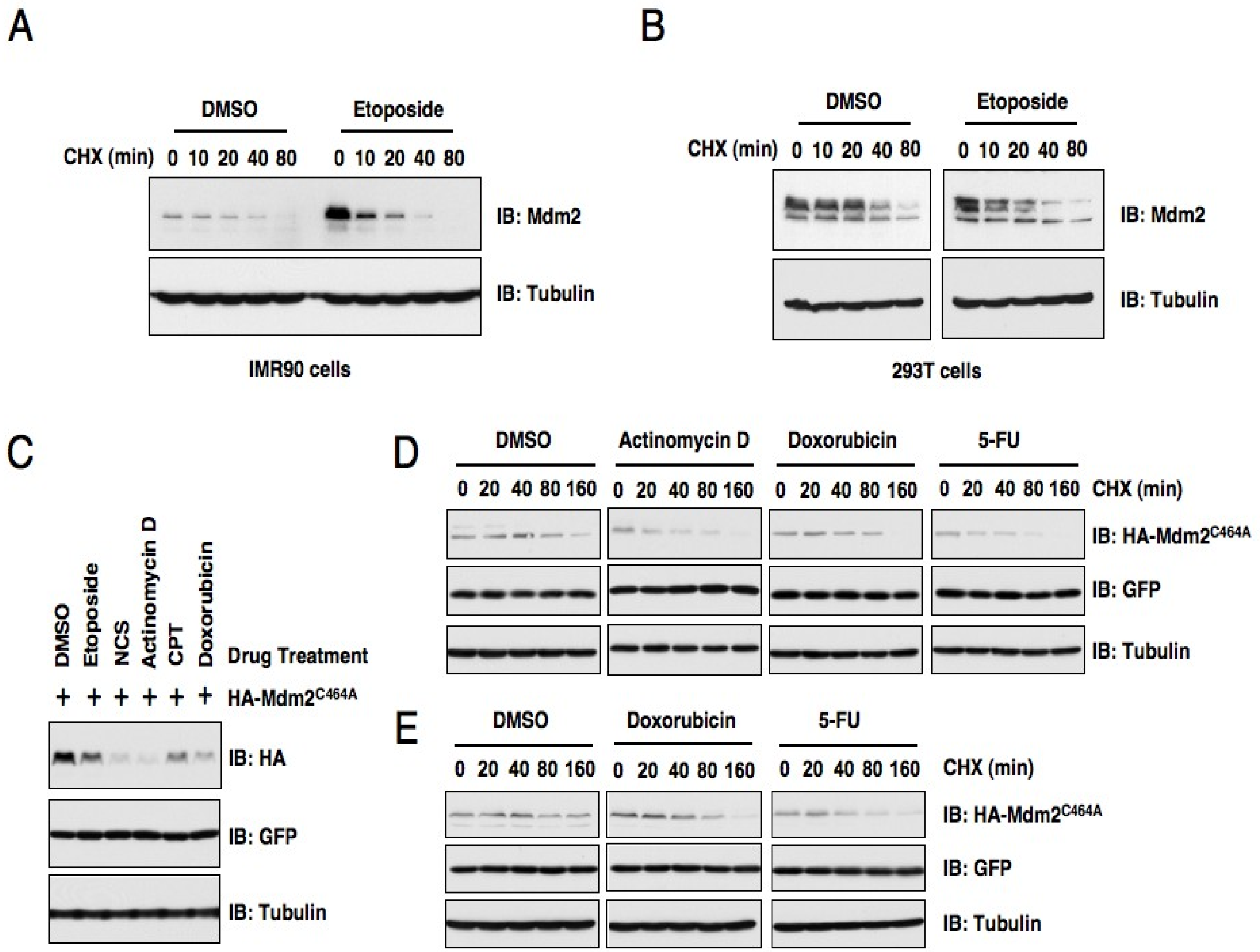

Supplementary Figure 1: DNA damage destabilizes Mdm2.

A-B. IMR90 (**A**) and 293T (**B**) cells were treated with 25 μM etoposide for 1.5 hours before 20 μg/ml cycloheximide was added. At the indicated time points, whole cell lysates were prepared and immunoblots were probed with the indicated antibodies.

C. HeLa cells were transfected with the C464A mutant HA-Mdm2 plasmid. Twenty hours post-transfection, cells were split into 60 mm dishes, and after another 20 hours, treated with the indicated DNA damaging drugs for 3 hours. Afterwards, whole-cell lysates were prepared and immunoblots were probed with the indicated antibodies.

D-E. HeLa cells (**D**) and 293T cells (**E**) were transfected with the C464A mutant HA-Mdm2 plasmid. Twenty hours post-transfection, cells were split into 60 mm dishes, and after another 20 hours, treated with the indicated DNA damaging drugs for 3 hours. Afterwards, cells were treated with 20 μg/ml cycloheximide. At the indicated time points, whole cell lysates were prepared and immunoblots were probed with the indicated antibodies.

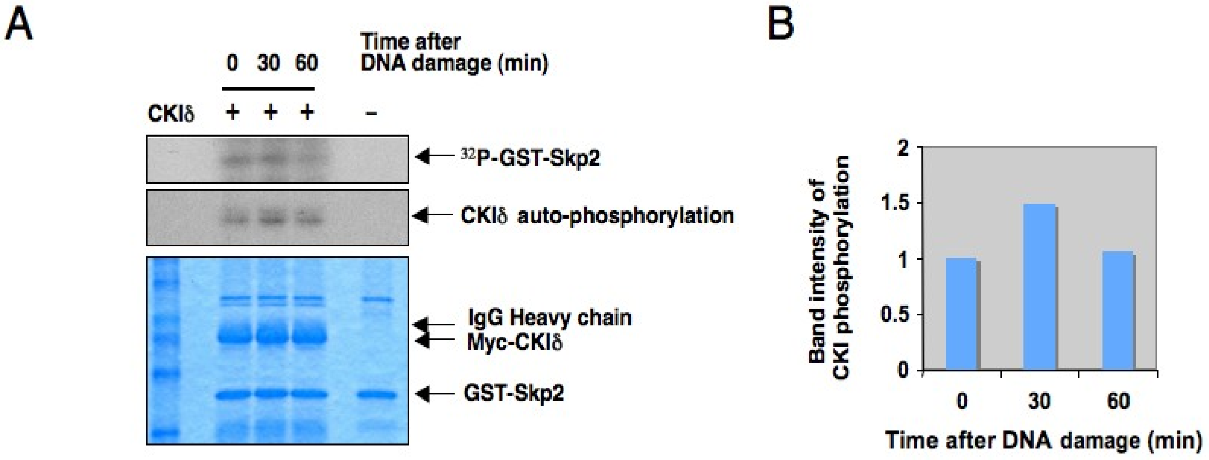

Supplementary Figure 2: DNA damage does not lead to a significant induction in CKIδ kinase activities.

A. Detection of CKIδ kinase activity changes in response to DNA damage by *in vitro* kinase assay. CKIδ kinase was purified by CKIδ-immunprecipitation from U2OS cells after treatment with etoposide for the indicated times, and then incubated with 5 μg of the GST-Skp2 protein in the presence of γ-^{32}P-ATP. The kinase reaction products were resolved by SDS-PAGE and phosphorylation was detected by autoradiography.

B. Quantification of the band intensities in **A**. The CKIδ auto-phosphorylation band intensity was normalized to Myc-CKIδ immunoprecipitation band, then normalized to the t=0 controls.
